# Nanostructuring of GeTiO amorphous films by pulsed laser irradiation

**DOI:** 10.3762/bjnano.6.92

**Published:** 2015-04-07

**Authors:** Valentin Serban Teodorescu, Cornel Ghica, Adrian Valentin Maraloiu, Mihai Vlaicu, Andrei Kuncser, Magdalena Lidia Ciurea, Ionel Stavarache, Ana M Lepadatu, Nicu Doinel Scarisoreanu, Andreea Andrei, Valentin Ion, Maria Dinescu

**Affiliations:** 1 National Institute of Materials Physics, 105 bis Atomistilor Street, 077125 Bucharest-Magurele, Romania; 2 Academy of Romanian Scientists, Bucuresti 050094, Romania; 3 National Institute of Plasma Lasers and Radiation, 409 Atomistilor Street, 077125 Bucharest-Magurele, Romania

**Keywords:** fast diffusion, GeTiO film, nanostructuring, pulsed laser annealing, cross-sectional transmission electron microscopy (XTEM)

## Abstract

Laser pulse processing of surfaces and thin films is a useful tool for amorphous thin films crystallization, surface nanostructuring, phase transformation and modification of physical properties of thin films. Here we show the effects of nanostructuring produced at the surface and under the surface of amorphous GeTiO films through laser pulses using fluences of 10–30 mJ/cm^2^. The GeTiO films were obtained by RF magnetron sputtering with 50:50 initial atomic ratio of Ge:TiO_2_. Laser irradiation was performed by using the fourth harmonic (266 nm) of a Nd:YAG laser. The laser-induced nanostructuring results in two effects, the first one is the appearance of a wave-like topography at the film surface, with a periodicity of 200 nm and the second one is the structure modification of a layer under the film surface, at a depth that is related to the absorption length of the laser radiation. The periodicity of the wave-like relief is smaller than the laser wavelength. In the modified layer, the Ge atoms are segregated in spherical amorphous nanoparticles as a result of the fast diffusion of Ge atoms in the amorphous GeTiO matrix. The temperature estimation of the film surface during the laser pulses shows a maximum of about 500 °C, which is much lower than the melting temperature of the GeTiO matrix. GeO gas is formed at laser fluences higher than 20 mJ/cm^2^ and produces nanovoids in the laser-modified layer at the film surface. A glass transition at low temperatures could happen in the amorphous GeTiO film, which explains the formation of the wave-like topography. The very high Ge diffusivity during the laser pulse action, which is characteristic for liquids, cannot be reached in a viscous matrix. Our experiments show that the diffusivity of atomic and molecular species such as Ge and GeO is very much enhanced in the presence of the laser pulse field. Consequently, the fast diffusion drives the formation of amorphous Ge nanoparticles through the segregation of Ge atoms in the GeTiO matrix. The nanostructuring effects induced by the laser irradiation can be used in functionalizing the surface of the films.

## Introduction

Laser pulse processing of surfaces and thin films is a useful tool for purposes such as the amorphous thin films crystallization [1–6], surface nanostructuring [7–10], laser-induced thin film dewetting [11–12], phase transformation and modification of physical properties of thin films [13–16]. The laser fluence values used for these applications are below the ablation threshold of the irradiated material in order to prevent a loss of material during laser processing.

The absorption length of ultraviolet (UV) laser radiation is in the range of tens of nanometers for many materials [17]. The laser pulse energy is deposited in a very thin layer beneath the surface of the laser target, which has a thickness of the order of magnitude of the laser absorption length. If the irradiated film thickness is greater than the laser radiation absorption length, the laser annealing takes place only in a surface layer of the film, and a gradual modification of the nanostructure or a crystallization can be induced in the film [18–19].

The surface heating of the film during the laser pulse action can be estimated if the physical properties of the films are known. The most important are the absorption coefficient of the laser radiation in the films and the heat diffusivity. Some of these parameters can be considered to be similar to those in bulk material, but in many cases of amorphous or multi-component films there are no corresponding bulk materials. The laser radiation absorptivity for many amorphous films is not known. Even in the case when the properties can be optically measured, they change at the beginning of each laser pulse, i.e., every successive laser pulse will see a different surface nanostructure with different absorptivity and different thermal diffusivity. Finally, the surface structure stabilizes after a number of laser pulses, which generally happens in the case of films crystallization. In addition to the thermal effect of the UV absorption in the target, the UV radiation can also induce photonic effects, such as the diffusivity enhancement of atomic species due to the lattice bonds softening [20–22] in the strong field of the laser pulse. High values of atomic diffusivity were observed for Ge diffusion in the case of laser crystallization annealing of SiGe amorphous film [18].

In this paper, we report about the nanostructuring at the surface and under the surface of amorphous GeTiO films by laser pulse action. The cross sectional study gives evidence of a fast diffusion effect, i.e., the formation of amorphous Ge nanoparticles through the segregation of Ge atoms in the GeTiO matrix.

## Experimental

Amorphous GeTiO films with a thickness of 330 nm were deposited by RF magnetron sputtering on Si(100) wafer substrates using Ge:TiO_2_ with 50:50 atomic ratio. Details on the film deposition are found in [23]. These GeTiO amorphous films were irradiated with laser fluences from 10 to 30 mJ/cm^2^ and different numbers of laser pulses in the range from 10 to 500. The laser irradiations were performed by using the fourth harmonic (λ = 266 nm) radiation of a Nd:YAG laser (Surellite II, "Continuum", USA) working in TEM00 mode, giving a maximum pulse energy of 100 mJ for the fourth harmonic with a pulse length of 5–7 ns and an adjustable repetition rate of 1–10 Hz. The experimental value of the laser average fluence is measured with an energymeter (Gentec QE 65 LP, Noise Level Energy 10 µJ, maximum frequency 100 Hz).

The laser irradiations were performed in air, perpendicularly to the film surface, using the central part of the laser beam, with a diameter of 7 mm having a rather homogeneous intensity. However, at the micrometer scale the laser beam is not homogeneous, because the high coherence of the Nd:YAG laser radiation gives rise to interference effects on the target surface, which induces local intensification of the laser fluence. Under these conditions, the film surface temperature during the laser pulse duration can only be estimated as an average value.

The nanostructuring of the amorphous GeTiO films starts at very low laser fluences, and the evolution of their morphology cannot be considered as a sign of melting. All laser treatments were conducted at low fluences, so that the films remain practically in the solid state phase, as the surface temperature estimation shows.

Before and after laser irradiation, the nanostructure of the film surfaces was observed by scanning electron microscopy (SEM) and atomic force microscopy (AFM). The structure in the depth of the irradiated films was studied by cross-section transmission electron microscopy (XTEM). The specimens for XTEM were prepared by the conventional method: Small pieces cut from the irradiated area of the film sample were glued face to face, followed by mechanical polishing and ion milling in a Gatan PIPS model 691 aparatus. Transmission electron microscopy was performed by using a Jeol ARM 200F electron microscope, performing normal TEM imaging, scanning transmission electron microscopy-high angle annular dark field (STEM-HAADF) imaging and energy-dispersive X-ray spectroscopy (EDX). The estimation of the film surface temperature was performed by using the Heat Flow software [24].

## Results

**Figure 1:**
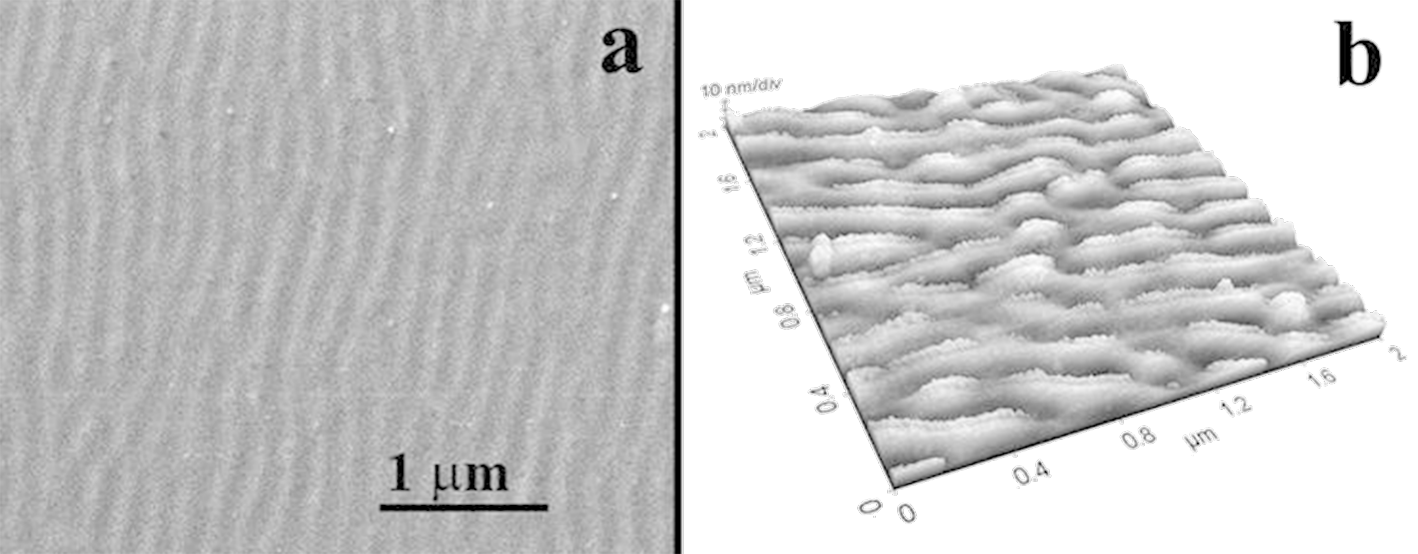
Surface relief of the GeTiO film after laser irradiation with 100 laser pulses at 15 mJ/cm^2^ fluence. (a) SEM image and (b) AFM image.

A quasi-coherent wave relief with a periodicity of about 200 nm and 10 nm amplitude was observed on the surface of the GeTiO film, after irradiation with 100 laser pulses with a fluence of 15 mJ/cm^2^. This periodicity is smaller than the 266 nm wavelength of the laser radiation. Figure 1 shows the SEM (Figure 1a) and AFM (Figure 1b) images of the irradiated surface area of the film.

**Figure 2:**
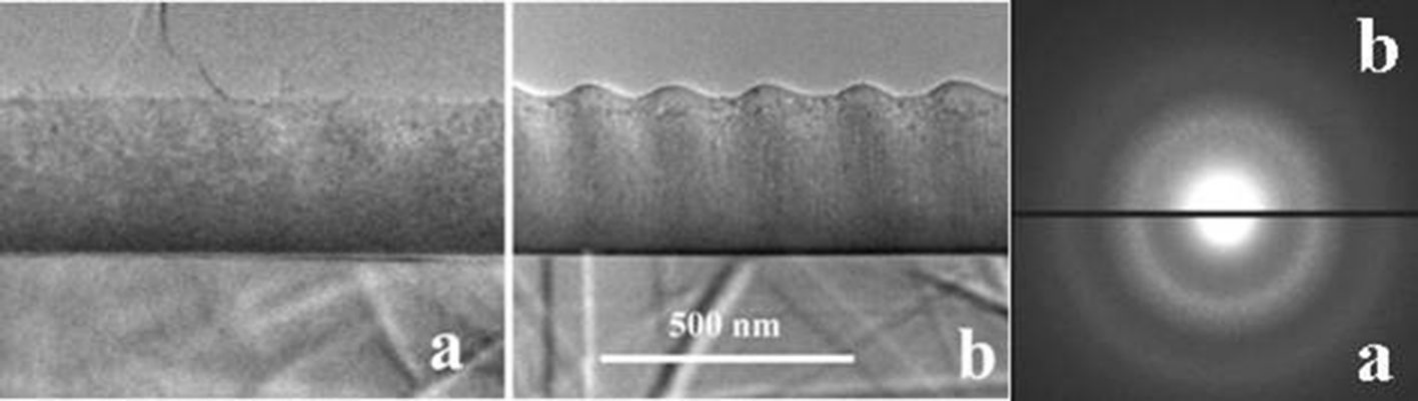
Low-magnification XTEM images of the amorphous GeTiO film, before (a) and after (b) laser irradiation at low fluence (15 mJ/cm^2^). A small, but evident, increase of the film volume happens. The film structure remains amorphous after laser irradiation, as can be seen from the corresponding SAED patterns.

Figure 2 shows low-magnification XTEM images of the film before (Figure 2a) and after (Figure 2b) laser irradiation with 100 pulses at low laser fluence (15 mJ/cm^2^). The wave relief is visible in the XTEM specimen because the film sample was cut perpendicular to the wave relief formed on the film surface. The film structure remains amorphous after laser irradiation, but a small increase of the volume is observed at the film surface due to the laser pulse action.

**Figure 3:**
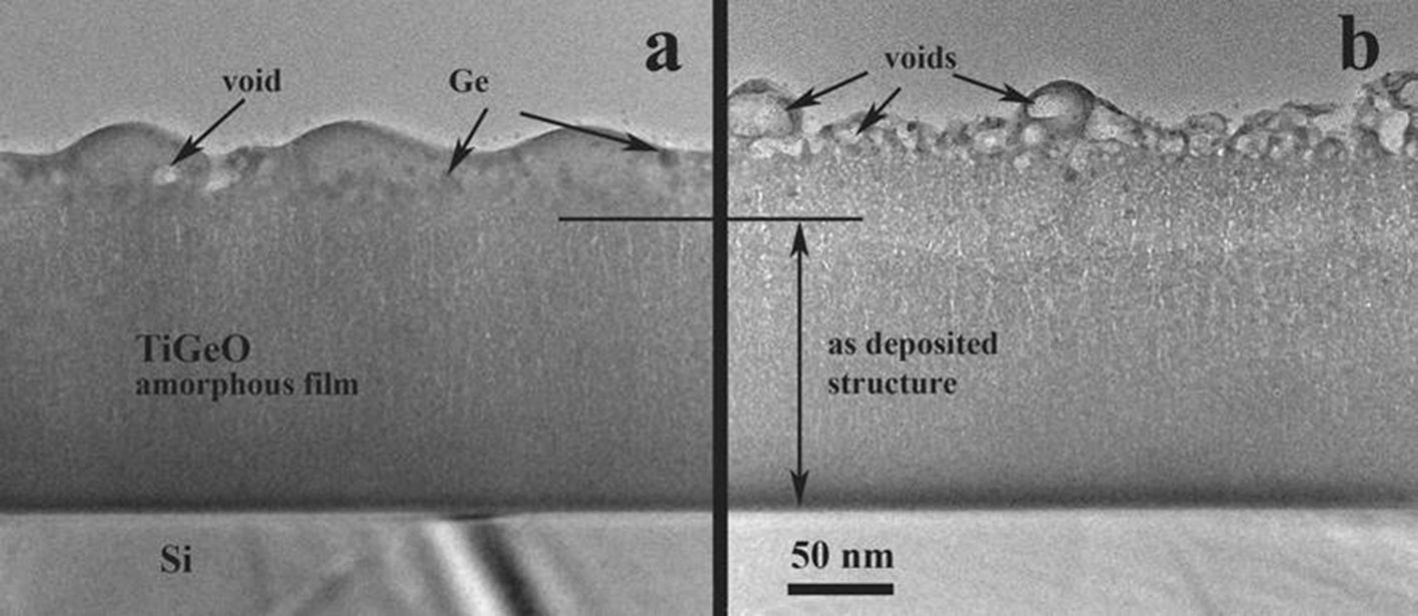
XTEM images of the amorphous GeTiO RF film after laser irradiation with 266 nm laser radiation. (a) Low fluence (15 mJ/cm^2^) and (b) higher laser fluence (20 mJ/cm^2^). The film structure morphology is transformed in a depth of about 50 nm.

The morphology and the structure of the modified film surface layer strongly depend on the laser fluence (Figure 3a and Figure 3b). At a higher fluence (20 mJ/cm^2^), the wave relief disappears and an irregular structure of nanovoids appears in the surface layer. A closer look at the transformed surface layer (see Figure 3) reveals the formation of spherical nanoparticles and nanovoids. The formed nanovoids contribute to the small volume increase even at 15 mJ/cm^2^ (Figure 3a).

**Figure 4:**
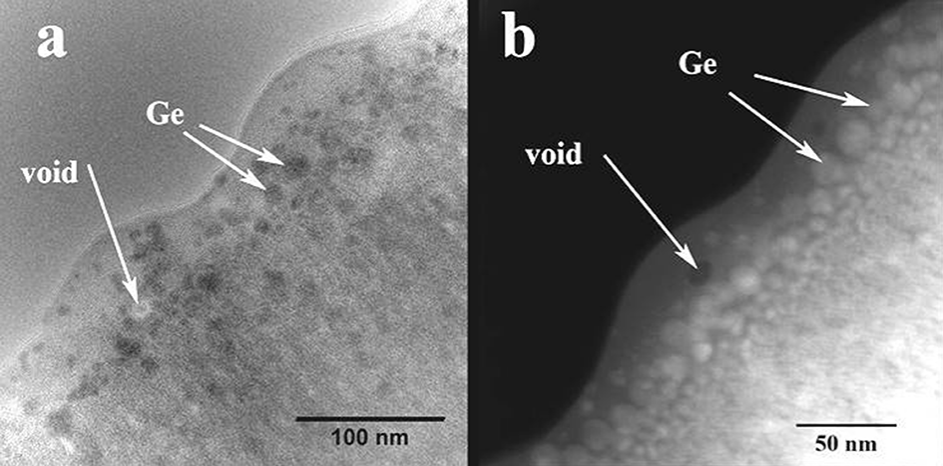
Morphology details of the GeTiO film surface layer structure revealed by the cross sectional observations. (a) XTEM conventional image and (b) a similar area viewed by STEM-HAADF method.

The most interesting transformation happened beneath the film surface, as revealed by detailed observations (Figure 4). The film structure is modified over a depth of about 50 nm and all the rest of film remains unchanged. The structure of the modified layer is also amorphous.

**Figure 5:**
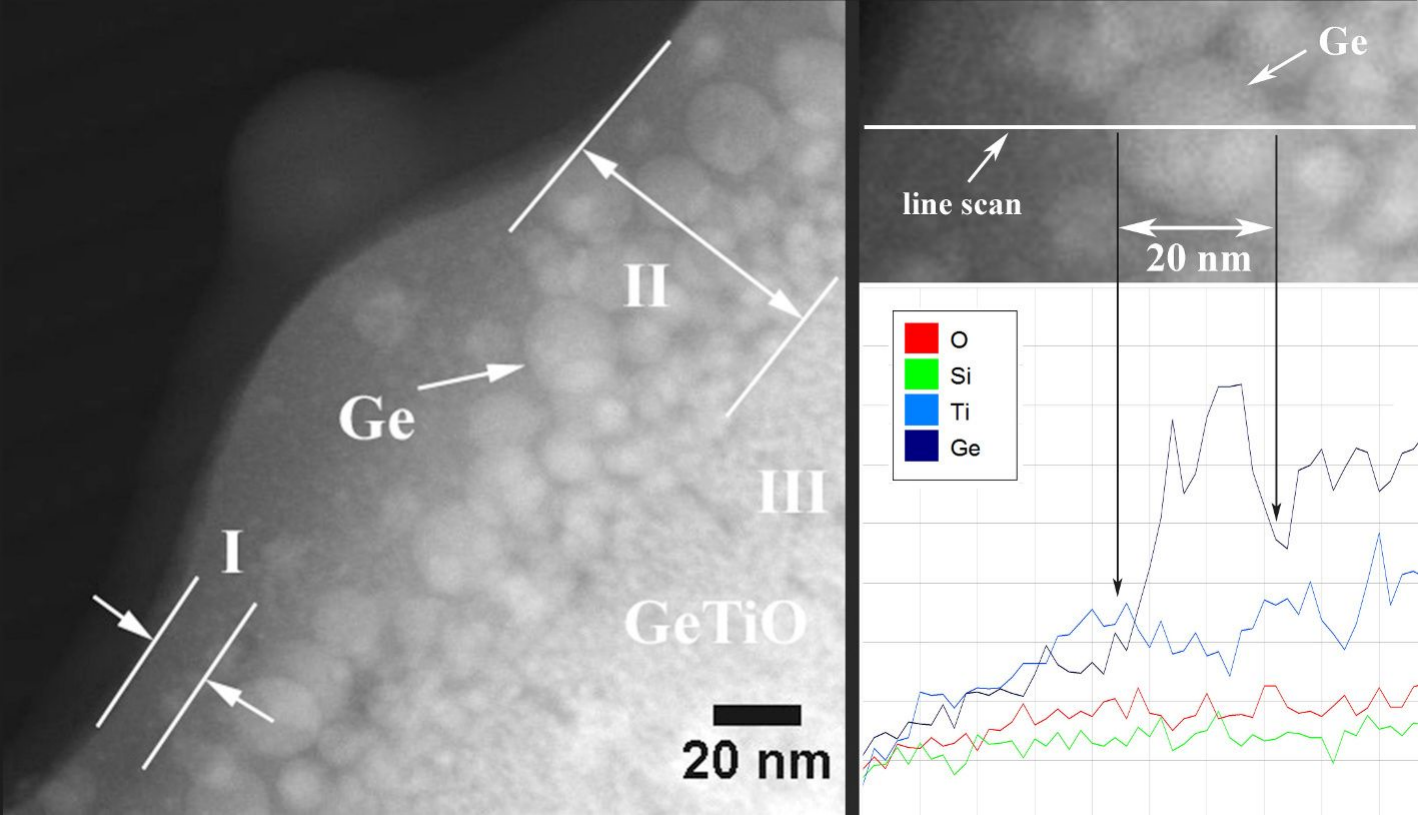
STEM-HAADF image (left) and a detail from the same image correlated with line scan EDX analyse (right) performed on the XTEM specimen prepared from the GeTiO film irradiated with 100 laser pulses at a fluence of 15 mJ/cm^2^.

During laser pulse irradiation, the morphology of the film surface layer affected by the laser pulse actions gradually changes. Spherical amorphous Ge nanoparticles are formed by Ge atoms segregation. These spherical Ge nanoparticles have 5 nm diameters at the interface with the region of the unchanged amorphous film structure (region III in Figure 5) and grow up to about 20 nm in the middle of the laser-transformed layer (region II in Figure 5). The first 10 nm layer under the film surface (region I in Figure 5) contains less Ge and only very few spherical Ge nanoparticles, as it can be observed from the HAADF-STEM images in Figure 4b and Figure 5.

**Figure 6:**
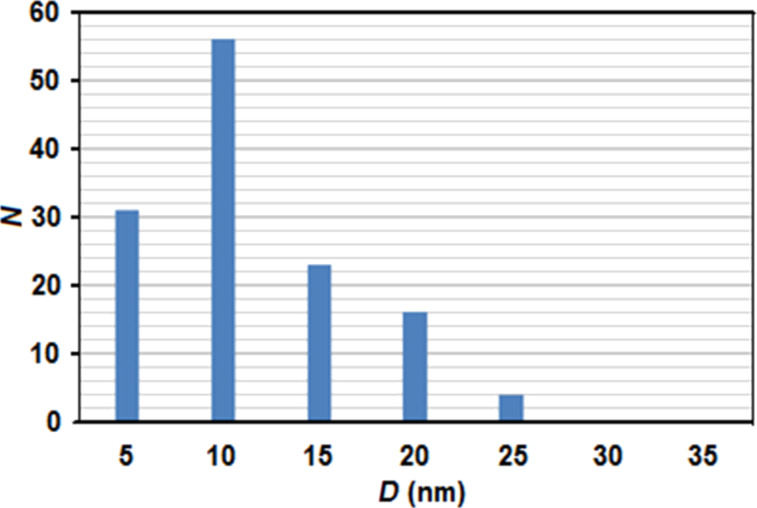
Size distribution of the Ge nanoparticles in region II of the laser transformed layer. The distribution is made from 130 nanoparticles counted on STEM images in which the Ge content is revealed by the *Z*-contrast. The average size is 11.5 nm.

The EDX line scan analysis, performed along the green line shown in Figure 5, reveals the majority of Ge content of the amorphous spherical nanoparticle, arrowed in the image, where the Ge signal is much higher than the Ti signal. The spherical amorphous Ge nanoparticles formed at the interface with the non-modified amorphous film (interface between region II and region III), have a minimum size of 5 nm. The size grows to about 20 nm in the middle of the layer affected by laser (see region II in Figure 5). The size distribution of the Ge nanoparticles is shown in Figure 6. The size range is between 5 and 25 nm and the average size is 11.5 nm.

**Figure 7:**
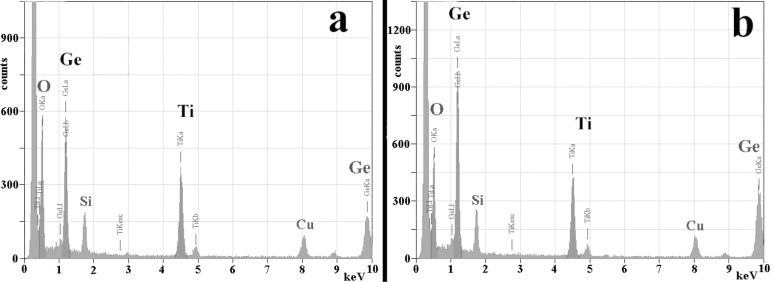
EDX spectra collected on the cross section specimen with an electron beam of 50 nm diameter. (a) Spectrum of the laser-modified layer, and (b) spectrum from a similar specimen area located in the middle of the GeTiO film, where the amorphous structure is not modified by the laser pulse action. The Cu signal comes from the Cu grid and Si peak is due to the redeposition during the ion thinning process.

The average composition of the modified layer and of the non-modified GeTiO film was measured by EDX using an electron spot-size of about 50 nm in diameter, which integrates the content of the total thickness of the laser transformed surface layer (see Figure 7).

The overall content of Ge in the modified top layer is less than in the middle of the film. So, in the modified surface layer, the Ge:Ti atomic ratio is between 2 and 2.3 depending on the site of measurement, while in the middle of the film it is close to 3 as it results from the RF magnetron sputtering films preparation. This shows that about 1/3 of the Ge content is lost from the surface layer affected by the laser radiation, and 2/3 of it can be segregated in amorphous Ge nanoparticles.

## Discussion

The nanostructure formed at the GeTiO film surface by pulsed laser irradiation shows that several effects are produced during the laser pulse action. The first one is the wave-like relief formed on the film surface, which has a periodicity of about 200 nm, less than the laser radiation wavelength. The second one is the segregation of the Ge nanoparticles under the film surface by a fast diffusion process in the surface layer related to the laser absorption depth.

The formation of periodic ripples due to laser pulse irradiation was already reported a long time ago [25–26], and in the case of perpendicular laser irradiation, the period of the ripple is equal to the laser wavelength or harmonics [27]. This happens at all wavelengths and pulse durations, as in the case of femtosecond laser irradiation [28].

The stress-induced periodic-ripples mechanism demonstrated for crystalline Si [29] could be used for amorphous materials if we define a stress yield for plastic flow. Such a mechanism can be imagined based on the shear transformation-zone theory of plastic deformation near the glass transition [30].

**Figure 8:**
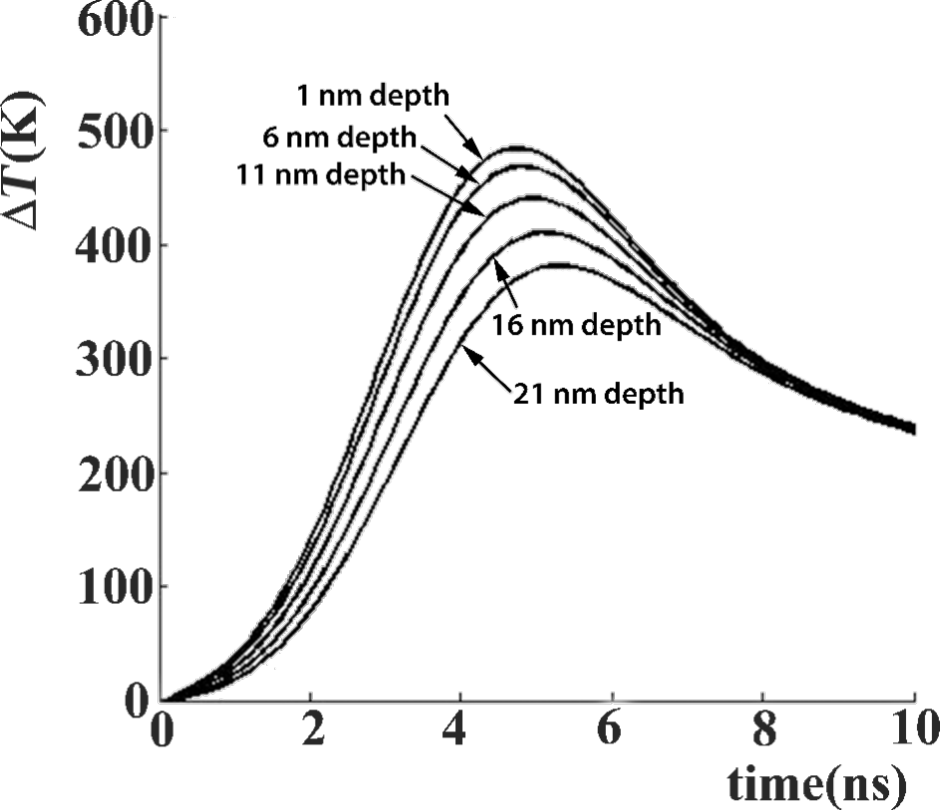
Temperature estimation for different depths beneath the GeTiO film surface during the laser pulse action, considering a Gaussian shape of the laser pulse with a total duration of 7 ns and a laser fluence of 30 mJ/cm^2^.

The temperature due to the laser heating was estimated by using the Heat Flow software [24]. Figure 8 shows the temperature variation at different depths of the surface layer during the laser pulse action. For the calculation, the physical parameters were set to maximize the temperature. The absorption coefficient used is 10^6^ cm^−1^ (similar to Ge [17]), and the heat diffusivity is similar to that of TiO_2_ (0.02 cm^2^/s). Even under these conditions, the temperature grows up to about 500 °C for a laser fluence of 30 mJ/cm^2^, which is about double of the average fluence measured for the laser beam. Under these conditions, it is clear that the film surface does not melt during the laser pulse action. The melting temperature for Ge is about 900 °C [17], and of GeO_2_ and TiO_2_ is much higher [31].

However, the wave relief formed at the surface at low laser fluence, reveals a possible viscous character of the film surface, which probably happens in amorphous films in which a glass transformation process could happen [32]. For amorphous Ge, the temperature of glass transformation is around 600 °C [33], and probably the GeTiO structure has a similar behaviour. The glass transition of the GeTiO amorphous structure can be triggered under the laser pulse action.

At higher laser fluences, the wave relief disappears and a many small spherical (20–30 nm) nanovoids and a few larger ones (40–50 nm) appear at the film surface. We consider that these nanovoids are formed by the aggregation of the molecular GeO gas produced in the film matrix, during laser beam action. GeO gas can be formed around 600 °C [34].

The formation of Ge nanoparticles in a dielectric matrix was also evidenced in amorphous GeTiO films annealed in a conventional furnace [23]. The annealing at about 600 °C leads to the formation of Ge nanocrystals in the film matrix, which is formed by a crystallized mixture of two phases, the Ge in TiO_2_ anatase phase and the rutile phase (Ti in GeO_2_ rutile phase). If annealing is performed at 700 °C, the (GeTi)O_2_ rutile phase decomposes and a layer of GeO_2_ nanocrystallites appears on the film surface. This clearly shows that Ge diffuses out of the film as GeO.

In the case of laser annealing (irradiation), a fraction of Ge escapes from the top surface layer as revealed by the EDX measurements. We consider that the thickness of the surface layer which contains less Ge nanoparticles (zone I in Figure 4) reveals the diffusion length of Ge species during laser pulse annealing. In the inner zone II, the fast diffusion of Ge under the laser pulse field induces the formation of the amorphous Ge spherical nanoparticles. The Ge nanoparticles formation can be explained only by assuming the fast diffusion of Ge in a surface layer with its thickness being related to the laser radiation absorption length. The laser pulse duration is τ = 7 ns, and the normal diffusivity of the Ge species in the solid state phase is of the order of *D* = 10^−14^ m^2^/s at temperatures of about 900 °C [35]. For a total duration of 100 pulses the total diffusion length of the Ge species can be of the order of (*D*·τ)^1/2^ = 10 pm. Normally, a diffusion or segregation of Ge atoms in the solid phase cannot be expected. However, the experimental data show that the diffusion length of Ge atoms is about 10 nm during the laser pulse action, corresponding to a diffusivity in the range of 10^−5^ to 10^−6^ m^2^/s, which is typical for the liquid phase. In the viscous phase the diffusivities are much smaller than in the liquid phase as the fraction of the broken bonds in the viscous phase is much smaller than in the liquid phase. The fast diffusion of Ge in the solid matrix was also evidenced in the case of laser irradiation of amorphous SiGe films [18].

**Figure 9:**
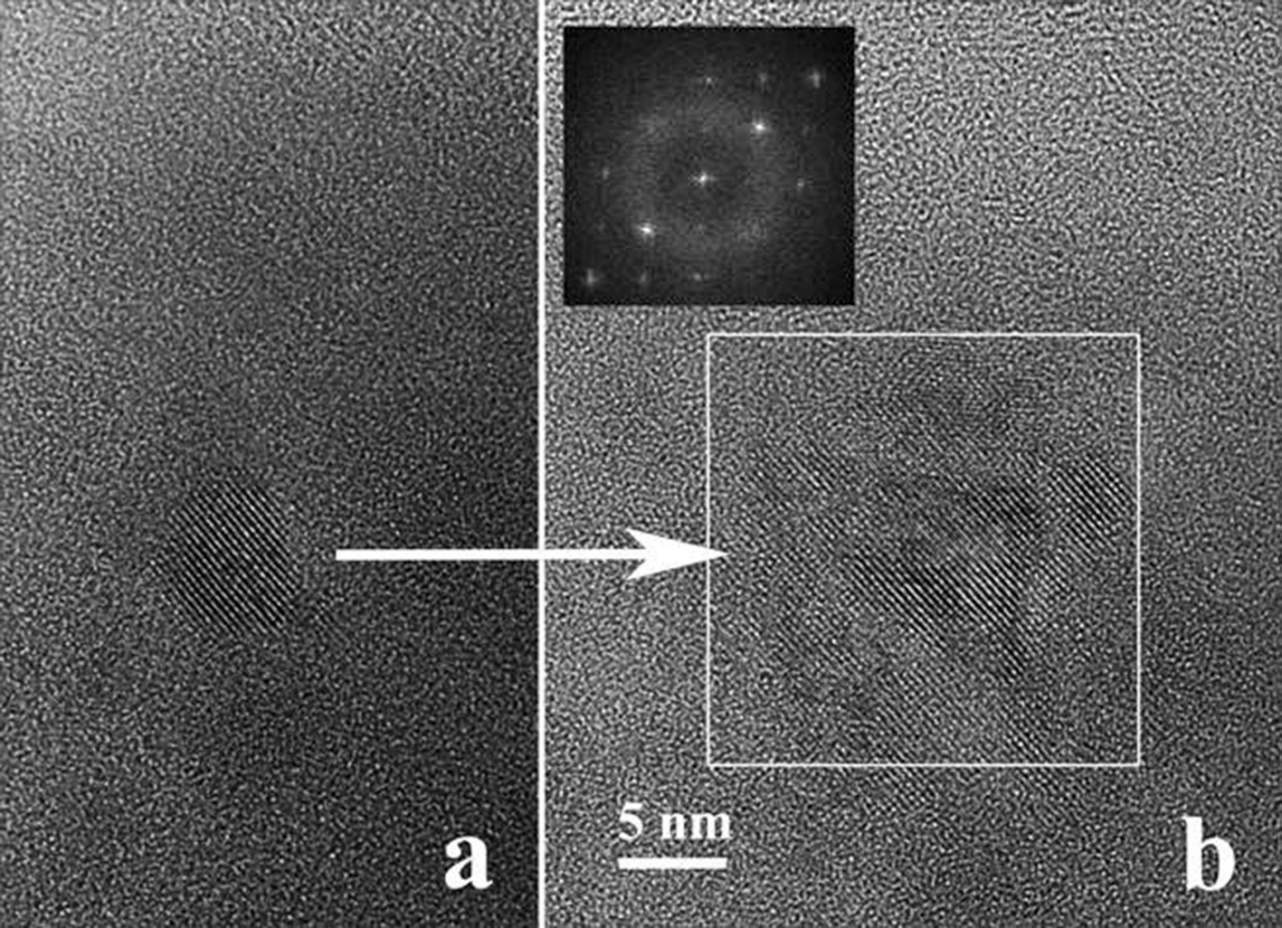
Crystallization (a) and subsequent crystal growth (b) of the Ge amorphous nanoparticle under the high density electron beam irradiation in the microscope. The FFT inserted in the (b) shows a pattern characteristic for the (GeTi)O_2_ rutile structure.

The prolonged high-resolution TEM observation of the amorphous spherical Ge nanoparticles situated in the very thin areas of the XTEM specimen induces local heating and the crystallization of the amorphous Ge nanoparticles. Figure 9a shows such a spherical Ge nanoparticle crystallized during observation. If the electron irradiation continues for several minutes, the spherical crystallite becomes larger through a crystal growth process, as shown in Figure 9b.

**Figure 10:**
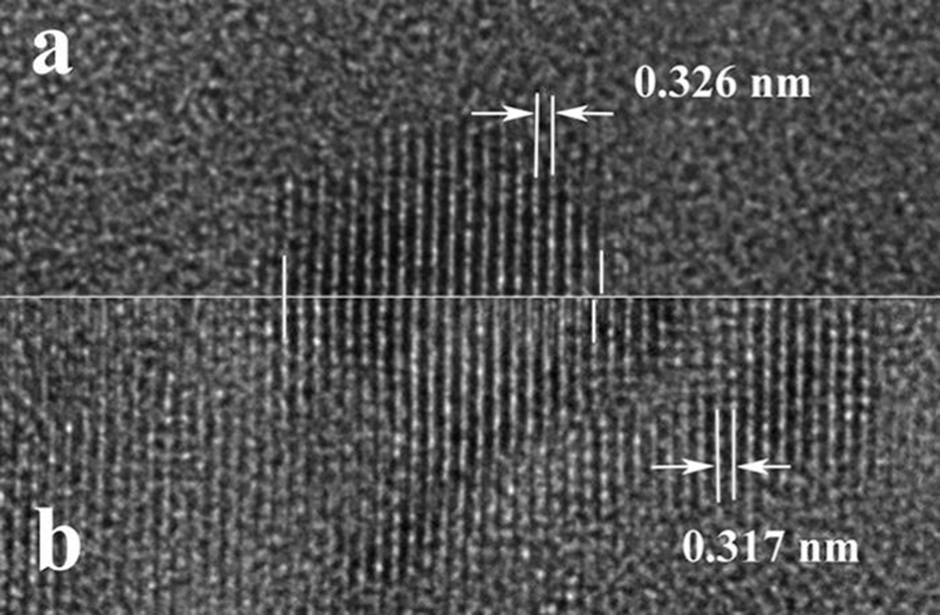
High-resolution TEM images comparing details of the initial crystallized particle (a) and the same area of the particle after the crystal growth process (b). The lattice fringe spacing in panel (b) is smaller than that in panel (a).

The fast Fourier transform (FFT) pattern inserted in Figure 9b shows a quadratic structure for the larger crystallite. By comparing the lattice fringes of the crystallized particle, before and after the crystal growth process (detail in Figure 10) we observe a contraction of the lattice fringe spacing from 0.326 to 0.317 nm, and a small rotation (about 2°) of the lattice fringes direction. The 0.326 nm lattice spacing is characteristic for the cubic Ge and the crystallized particle has the size of the initial spherical Ge amorphous particle. After the crystal growth process, the crystallized area becomes larger and the visible lattice spacing becomes 0.317 nm. The 0.317 nm lattice spacing suggests the formation of the (GeTi)O_2_ rutile structure [23].

The initial Ge amorphous nanoparticles are produced by laser annealing which in turn initiates the segregation of Ge atoms. This segregation can start around a Ge-rich cluster in the GeTiO amorphous matrix. The crystallization of the Ge phase under electron beam irradiation is an indirect proof that these Ge amorphous particles are practically formed only by Ge atoms. The subsequent crystal growth process developed after the Ge crystallization will use the surrounding GeTiO material which has a smaller Ge content. The high-resolution TEM observations show that the crystalline planes of the cubic Ge (111) with a spacing of 0.326 nm are parallel to the (110) planes of the (GeTi)O_2_ rutile structure having a spacing of 0.317 nm. This means that around the Ge spherical crystallite, the crystal growth process favours the growth of the rutile structure.

## Conclusion

We investigated the effects of nanostructuring produced by the pulsed-laser irradiation at the surface and beneath the surface of amorphous magnetron sputtered GeTiO films. The nanostructuring consists of two simultaneous processes, namely the laser-induced nanostructuring, i.e., the appearance of a wave-like topography on the film surface and the structure modification beneath the film surface, in a depth that is correlated with the laser radiation absorption length. The periodicity of the wave-like relief is smaller than the laser wavelength. The laser annealing of the amorphous GeTiO films results in an unusual segregation of Ge atoms leading to formation of amorphous Ge nanoparticles beneath the film surface due to the fast diffusion of Ge atomic species.

The laser irradiation at low fluences used in our experiments heats the film surface only up to several hundred degrees (less than 500 °C), which is far from the melting point of the amorphous matrix. A glass transition effect is expected in the amorphous GeTiO film, transforming the matrix in a viscous one. This is supposed to favor the formation of the wave relief at the film surface.

We show that the Ge segregation takes place through fast Ge diffusion, at diffusivity values typical for the liquid phase. The viscous behavior of the modified layer is not enough to explain the high Ge diffusivity.
